# Supplementary material for: Control of chrysanthemum flowering through integration with an aging pathway
Source: Nat Commun. 2017 Oct 10;8:829. doi: 10.1038/s41467-017-00812-0 (PMC5635119; doi:10.1038/s41467-017-00812-0)
Supplement: Supplementary file 1 — Supplementary Information [file 41467_2017_812_MOESM1_ESM.pdf]

Description of Supplementary Files

File Name: Supplementary Information
Description: Supplementary Figures and Supplementary Table.

File Name: Supplementary Data 1
Description: Differentially transcribed genes in *CmNF-YB8*-RNAi plants compared with wild type chrysanthemum.

File Name: Peer Review File

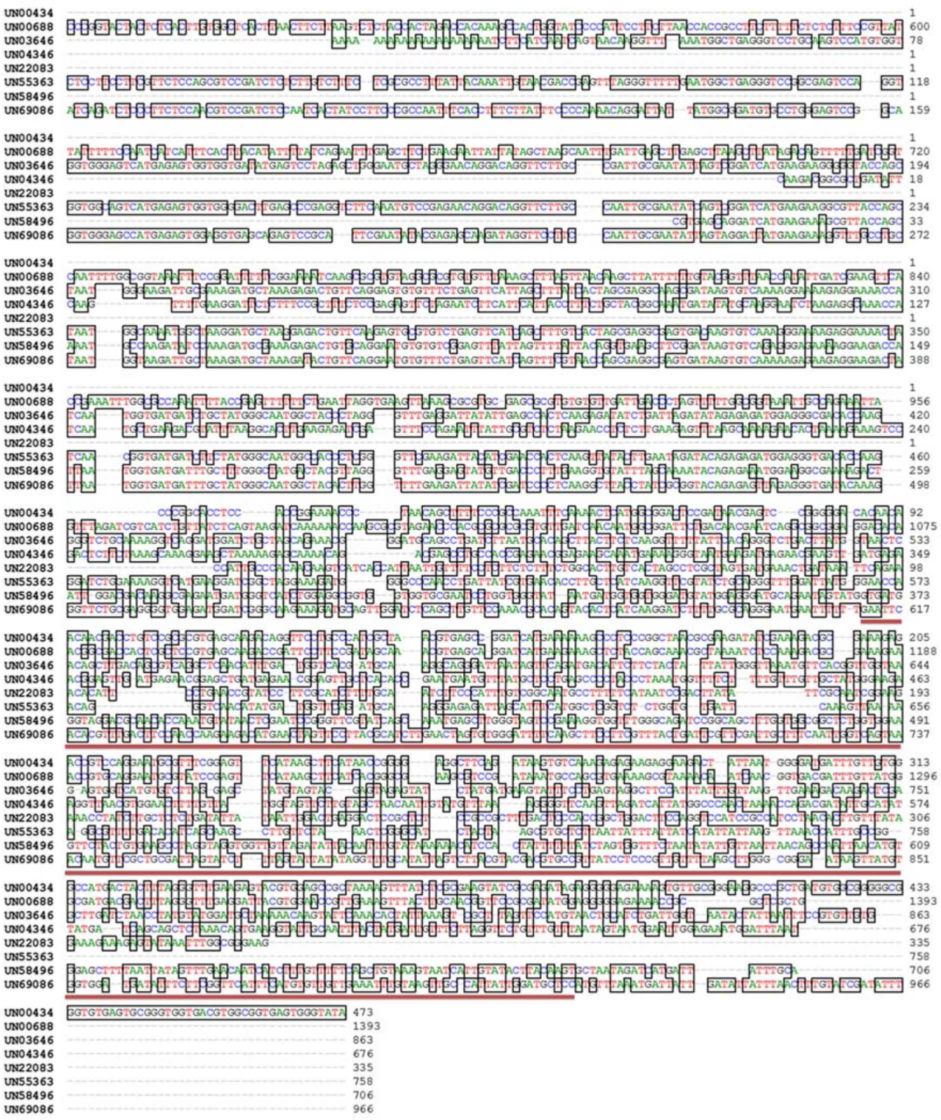

Supplementary Figure 1. Alignment of the cDNA sequences of eight chrysanthemum NF-YB family members. The region used for the RNAi silencing of *CmNF-YB8* (UN69086) is underlined in red. Chrysanthemum NF-YB family member sequences have been deposited in the NCBI database: UN00434 corresponds to *NFYB1* (KT253136) in NCBI; UN00688 corresponds to *NFYB2* (KT253137); UN04346 corresponds to *NFYB4* (KT253139); UN22083 corresponds to *NFYB6* (KT253141); UN58496 corresponds to *NFYB18* (KT253143).

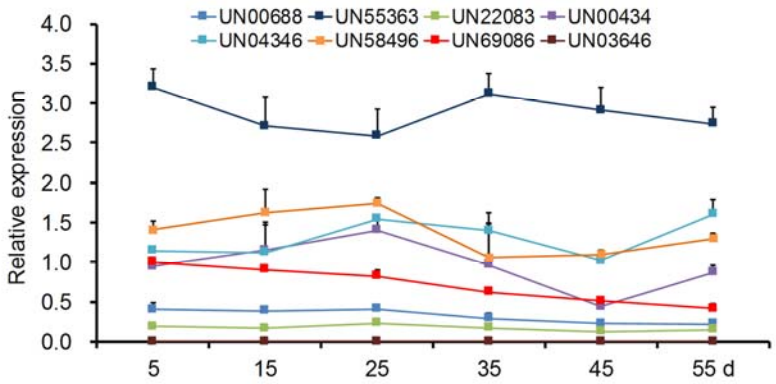

Supplementary Figure 2. Expression analysis of eight NF-YB family members in apical buds of chrysanthemum plants of different ages by qRT-PCR. Plants were grown under long day conditions. Samples were harvested every 10 d from 5 d after propagation. *UBIQUITIN* was used as the control. Three independent experiments were performed and error bars indicate standard deviation.

```
1      CGTCCGATCTCCAATCACTATCCTTCCCGCCAATTTCACCTTTCTTATTTCCCCAAAACAGGGTTATT
69     ATG GCG GAT GTG CCT GGG AGT CCG GCA GGT GGG AGC CAT GAG AGT GGA GGT GAG CAG AGT
        M   A   D   V   P   G   S   P   A   G   G   S   H   E   S   G   G   E   Q   S   20
129    CCG CAT TCG AAT ATA AGG GAG CAA GAT AGG TTC CTT CCG ATT GCG AAT ATT AGT AGG ATT
        P   H   S   N   I   R   E   Q   D   R   F   L   P   I   A   N   I   S   R   I   40
189    ATG AAG AAA GGT TTG CCT GCT AAT GGT AAG ATT GCT AAA GAT GCT AAA GAT ACT GTT CAG
        M   K   K   G   L   P   A   N   G   K   I   A   K   D   A   K   D   T   V   Q   60
249    GAA TGT GTT TCT GAG TTC ATC AGT TTC GTA ACC AGC GAG GCG AGT GAT AAG TGT CAA AAA
        E   C   V   S   E   F   I   S   F   V   T   S   E   A   S   D   K   C   Q   K   80
309    GAG AAG AGG AAG ACT ATT AAT GGT GAT GAT TTG CTA TGG GCA ATG GCT ACA CTT GGT TTT
        E   K   R   K   T   I   N   G   D   D   L   L   W   A   M   A   T   L   G   F   100
369    GAA GAT TAC ATC GAT CCC CTC AAG GCT TAC CTA TCG CGT TAC AGA GAG TTA GAG GGT GAT
        E   D   Y   I   D   P   L   K   A   Y   L   S   R   Y   R   E   L   E   G   D   120
429    ACA AAG GGT TCT GCG AGG GGT GGA GAT GGA TCG GGC AAG AAA GAT GCA GTT GGG TCT CAG
        T   K   G   S   A   R   G   G   D   G   S   G   K   K   D   A   V   G   S   Q   140
489    CTT GTT CCA AAC GCA CAG TAC ACT CAT CAA GGA TCT TTT GCG CAG GGA ATG AAT TTT TTG
        L   V   P   N   A   Q   Y   T   H   Q   G   S   F   A   Q   G   M   N   F   L   160
549    AAT TCA CAC GTTTGACTTCCAACCAAGAAGACACGAACTAGTTCCTCACGCATCTTTGAACTAGTGTGGGAT
        N   S   H   V
621 TTTCAAGCTTCCTTTGTTTACTGATTCGTTCGATTGGTTCAATTGGTCAGTAAACAATGTTCGCTGCGAT
691 TAGTATCTTTAGTATTATATAGGTTTGCATATTAGTCTTACGTACGACGTGCCGTTATCCTCCCGTTGTA
761 TTTAAGCTTGGGCGGGAATAAGTTATGTGGTGGATGATATTTCTTCGGTTCATTTCATGTGTTGTTGAAA
831 TTTGTAAGTTGTCATTATTGGATGCTCCATGTTTAAATGATTACTGATATTATTTAACTTTGTATCGATA
901 TTAGGTGTCGTTTGTTTTTTACAAAAAAAAAAAAAAAAAAAAAAAAAAAAAA
```

Supplementary Figure 3. CmNF-YB8 nucleotide and deduced amino acid sequences.

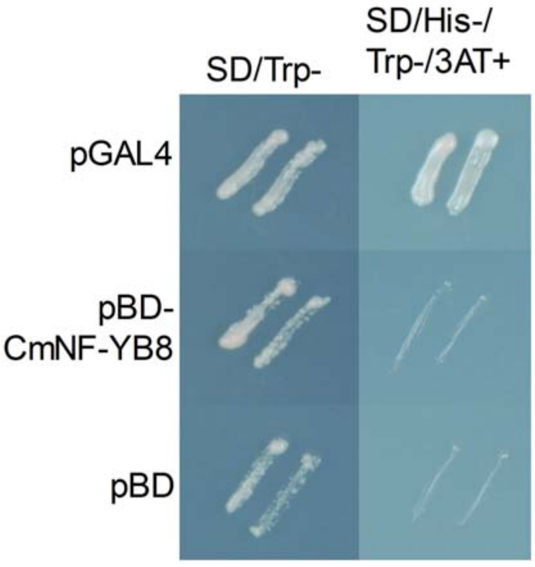

Supplementary Figure 4. Transactivation activity analysis of CmNF-YB8 in yeast cells. The pBD and pGAL4 plasmids were used as the negative and positive controls, respectively. SD/Trp-, SD medium without tryptophan; SD/His-/Trp-/3AT+ medium, SD medium without histidine and tryptophan, but with 3mM 3-AT. Three independent experiments were performed with similar results.

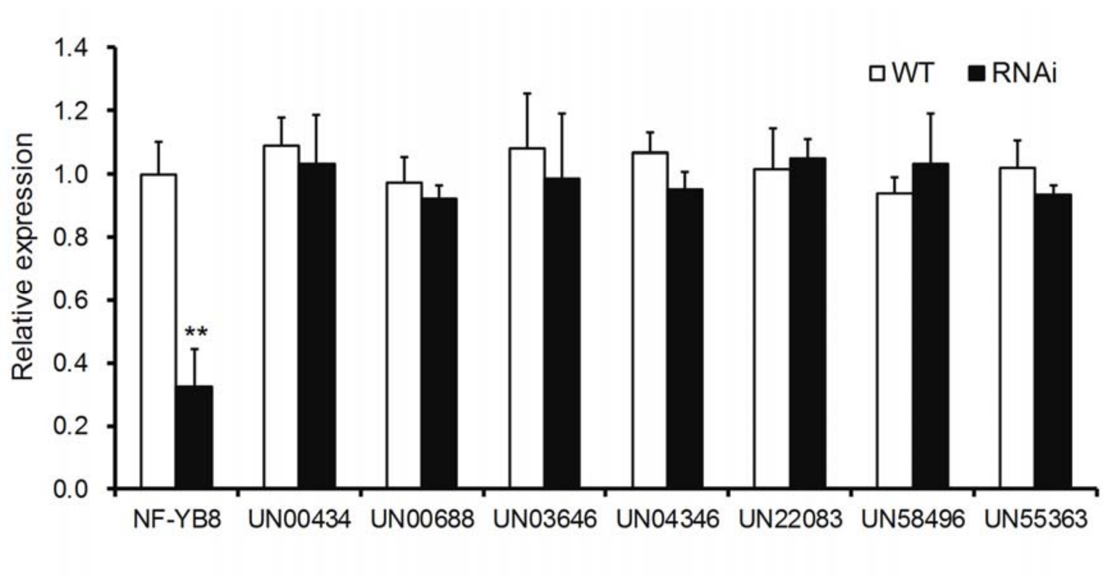

Supplementary Figure 5. Transcript abundance, determined by qRT-PCR, of chrysanthemum *NF-YB* genes in wild type (WT) and *CmNF-YB8*-RNAi plants (line 4). *UBIQUITIN* was used as the control gene. Three independent experiments were performed and error bars indicate standard deviation. Significant differences were determined using a Student's t-test (***P*<0.01).

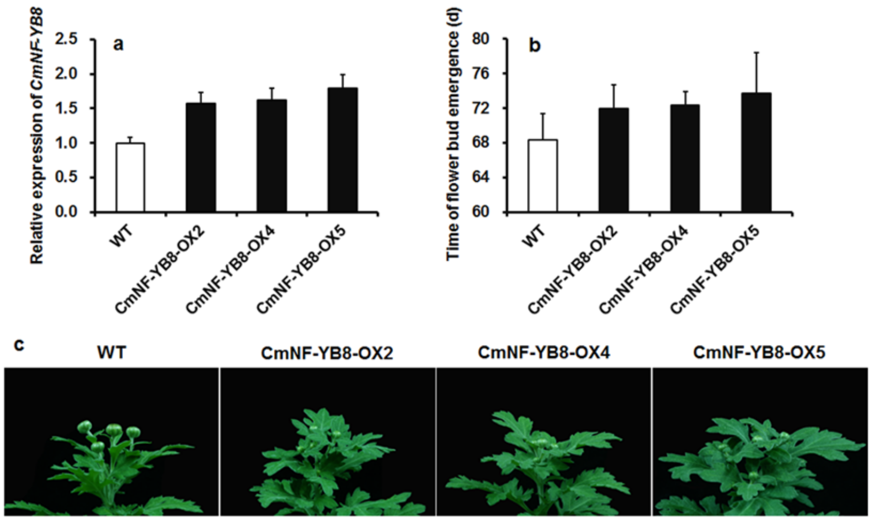

Supplementary Figure 6. The flowering time of *CmNF-YB8*-overexpressing chrysanthemum plants. (a) Transcript abundance of *CmNF-YB8* in wild type (WT) and *CmNF-YB8*-OX (overexpressing) plants determined by qRT-PCR. (b) The time of flower bud emergence in WT and *CmNF-YB8*-OX plants grown under short day (SD) conditions. (c) The generative phenotypes of WT and *CmNF-YB8*-OX plants were recorded. Three independent experiments were performed and error bars indicate standard deviation.

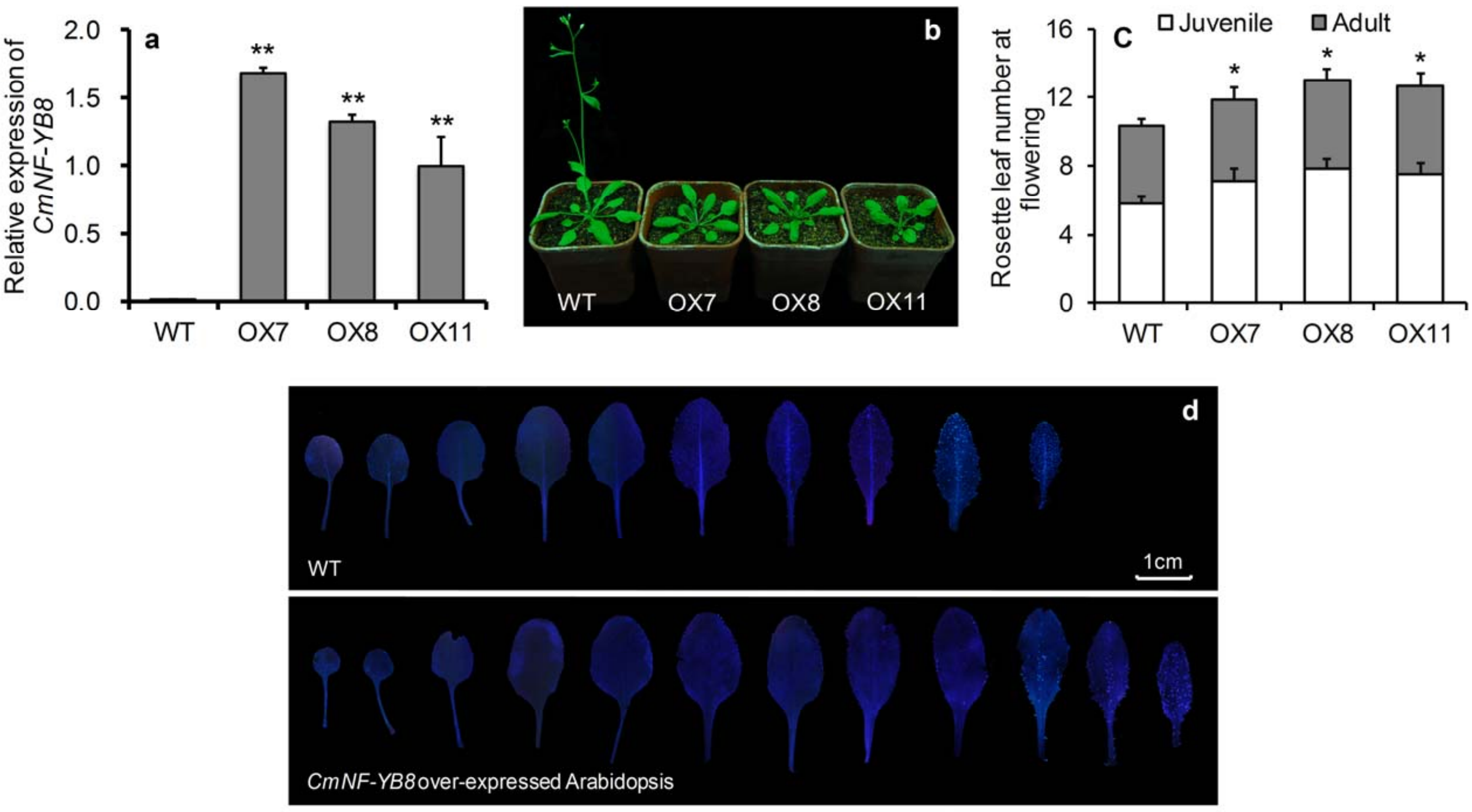

Supplementary Figure 7. The juvenile vegetative phase and the flower time of *CmNF-YB8*-overexpressing *Arabidopsis thaliana* lines. (a) Transcript abundance of *CmNF-YB8* in *CmNF-YB8*-overexpressing *A. thaliana*. (b) The generative phenotypes of wild type (WT) and *CmNF-Yb8*-overexpressing plants were recorded after 28 days growth under long day (LD) conditions. (c) Rosette leaf number of WT and *CmNF-YB8*-overexpressing lines at flowering time after growth under LD conditions. (d) The distribution of abaxial trichomes on leaves of WT and *CmNF-YB8*-overexpressing plants. Rosette leaves were stained with aniline blue and the auto-fluorescence of abaxial trichomes was visualized using ultra violet light excitation. OX7, OX8 and OX11 correspond to 3 independent *Super:CmNF-YB8*-GFP lines. Three independent experiments were performed and error bars indicate standard deviation. Asterisks indicate significant differences according to a Student's t-test (**P*<0.05, ***P*<0.01).

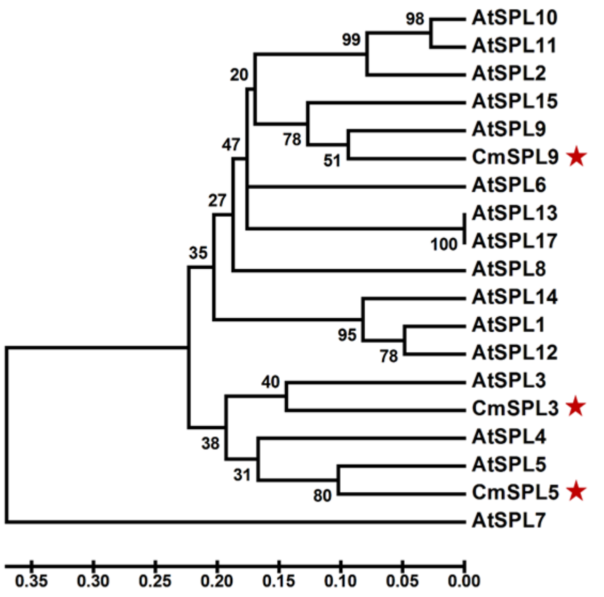

Supplementary Figure 8. Phylogenetic analysis of CmSPLs and *Arabidopsis thaliana* SPL proteins. Bootstrap values indicate the divergence of each branch, and the scale indicates branch length. *A. thaliana* SPL family sequences were obtained from the TAIR database: AtSPL1 (At2g47070), AtSPL2 (At5g43270), AtSPL3 (At2g33810), AtSPL4 (At1g53160), AtSPL5 (At3g15270), AtSPL6 (At1g69170), AtSPL7 (At5g18830), AtSPL8 (At1g02065), AtSPL9 (At2g42200), AtSPL10 (At1g27370), AtSPL11 (At1g27360), AtSPL12 (At3g60030), AtSPL13 (At5g50570), AtSPL14 (At1g20980), AtSPL15 (At3g57920), AtSPL17 (At5g50670).

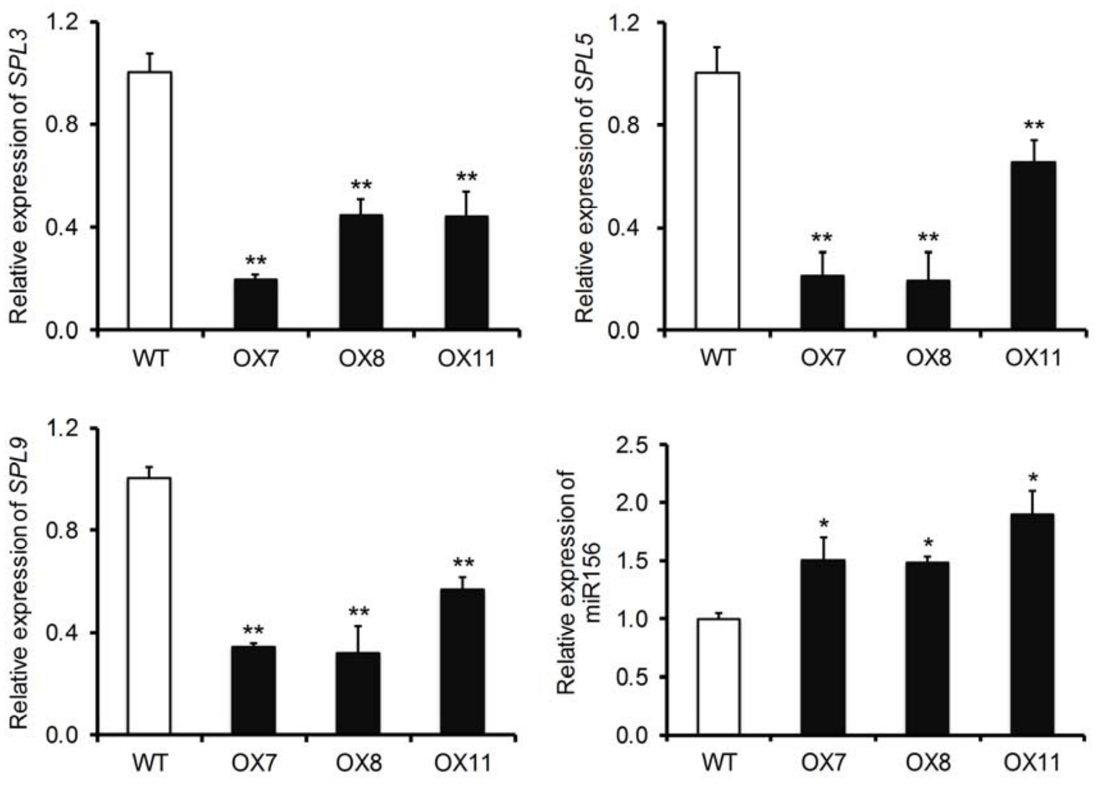

Supplementary Figure 9. Transcript abundance, determined by qRT-PCR, of *SPL* genes and miR156 in *CmNF-YB8*-overexpressing *Arabidopsis thaliana* lines. OX7, OX8 and OX11 correspond to 3 independent *Super:CmNF-YB8* lines. Three independent experiments were performed and error bars indicate standard deviation. Asterisks indicate significant differences according to a Student's t-test (*$P<0.05$, **$P<0.01$).

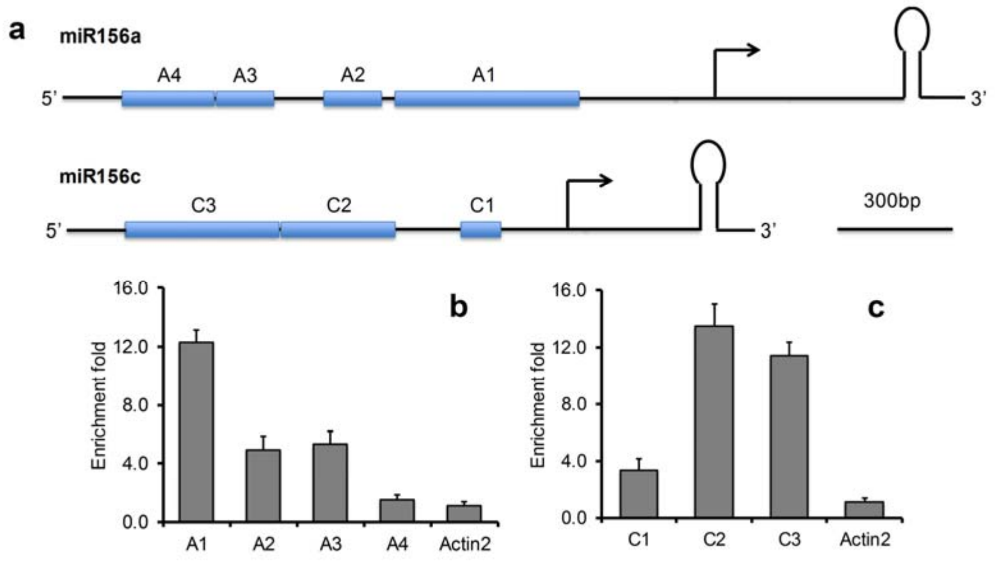

Supplementary Figure 10. ChIP-qPCR assay of CmNF-YB8 binding to the promoters of *MIRNA156A* and *MIRNA156C*. (a) Schematic representation of the upstream region of the fold-back structure of the miR156a and miR156c precursors. Blue boxes indicate the fragments amplified in the ChIP-PCR analysis. The major transcription start site (TSS) is denoted with a bent arrow. A1: -824~-351 bp, A2: -1034~-861 bp, A3: -1353~-1173 bp, A4: -1578~-1331 bp relative to major the TSS of *MIR156A*; C1: -298~-183 bp, C2: -759~-446 bp, C3: -1124~-724 bp relative to major the TSS of *MIR156C*. (b and c) ChIP-enrichment of indicated fragments in the *MIR156A* (b) and *MIR156C* (c) promoters. Chromatin from *Super:CmNF-YB8-GFP* expressing *A. thaliana* plants was immuno-precipitated with an anti-GFP antibody, and the amount of the indicated DNA in the immune complex was determined by quantitative PCR. An *Actin2* fragment was amplified as control. Three independent experiments were performed with similar results.

Supplementary Table 1. Primers used for this study.

	Primer set
	Forward and Reverse primer (5' -3')
For vector construction for overexpression	
CmYB8-PstI-F	5'-CTGCTGCAGATGGCGGATGTGCCTGGGAG-3'
CmYB8-SalI-R	5'-TGGTCGACAAACGTGTGAATTCAAAAAATTC-3'
For vector construction of chrysanthemum RNAi lines	
CmYB8-XhoI-F	5'-GAACTCGAGGAATTCACACGTTTGACTTCC-3'
CmYB8-KpnI-R	5'-CTGGTACCTGGAGCATCCAATAATGACAAC-3'
CmYB8-XbaI-F	5'-AATCTAGAGAATTCACACGTTTGACTTCC-3'
CmYB8-BamHI-R	5'-CTGGATCCTGGAGCATCCAATAATGACAAC-3'
For vector construction for transcriptional activation analysis	
CmYB8-EcoRI-R	5'-GACGAATTCAAACGTGTGAATTCAAAAAATTC-3'
CmYB8-XhoI-F	5'-CTGCTCGAGATGGCGGATGTGCCTGGGAG-3'
For vector construction for quantitative transient assay of miRNA targeting	
SPL3-F	5'-CTAGGGCGCTCTCTCTCTTCTGTCAA-3'
SPL3-R	5'-CCGGTTGACAGAAGAGAGAGAGCGCC-3'
SPL5-F	5'-CTAGGATGCTCTCTCTCTTCTGTCAA-3'
SPL5-R	5'-CCGGTTGACAGAAGAGAGAGAGCATC-3'
SPL9-F	5'-CTAGGGTGCTCTCTCTCTTCTGTCAA-3'
SPL9-R	5'-CCGGTTGACAGAAGAGAGAGAGCACC-3'
SPL_SNC-F	5'-CTAGGGTCTTGAGCTTGTTTTGCCAA-3'
SPL_SNC-R	5'-CCGGTTGGCAAAACAAGCTCAAGACC-3'
Spacer-F	5'-CTAGGATCGGTATCCAGCTCCCACAA-3'
Spacer-R	5'-CCGGTTGTGGGAGCTGGATACCGATC-3'
miR156a-XbaI-F	5'-CATCTAGAAAGGGTGATGACAGAAGAGAGT-3'
miR156a-SmaI-R	5'-CACCCGGGGAGGGGATGACAGATAGAGG-3'
For vector construction for yeast one-hybrid system	
Pro-miR156-F1	5'-TTTGATATTGGATCGGAATTCCGCTGATTTCATAACCCGCT-3'
Pro-miR156-1-F2	5'-TTTGATATTGGATCGGAATTCGGAAGACACACGTCACAC-3'
Pro-miR156-F2	5'-TTTGATATTGGATCGGAATTCATTTCATGTGCAGCGAACCA-3'
Pro-miR156-F3	5'-TTTGATATTGGATCGGAATTCAACCAGGCTTACTTTGACTTGA-3'
Pro-miR156-1-SpeI-R1	5'-CATCTGCAGACTAGTGTGACGTGTGTCTTCCCTC-3'
Pro-miR156-1-SpeI-R2	5'-CATCTGCAGACTAGTGTGTCTTAGTAGAATATGTAG-3'
Pro-miR156-SpeI-R2	5'-CATCTGCAGACTAGTGACTTGCTCAAGGATGGT-3'
Pro-miR156-SpeI-R3	5'-CATCTGCAGACTAGTTTAGAATACTCACAAGACGCACAAG-3'
Pro156-2-ccaat-mut-F	5'-CTACGTAAAAACGGTATAAAGTATAAC-3'
Pro156-2-ccaat-mut-R	5'-TACTTTATACCGTTTTTACGTAGTAT-3'
For vector construction for dual-luciferase reporter assay	
pGreen-CmYB8-BamHI-F	5'-GCGGGATCCATGGCGGATGTGCCTGGGA-3'
pGreen-CmYB8-KpnI-R	5'-CGGGGTACCTCAAACGTGTGAATTCAAAAAATTCAT-3'
Pro-miR156-1-BamHI-F2	5'-GCGGGATCCGGAAGACACACGTCACAC-3'
Pro-miR156-1-NcoI-R2	5'-CATGCCATGGTGAGTAACAAGTGGAGGTTAG-3'
Pro-miR156-2-ccaat-mut-F	5'-CTACGTAAAAACGGTATAAAGTATAAC-3'
Pro-miR156-2-ccaat-mut-R	5'-TACTTTATACCGTTTTTACGTAGTAT-3'
For qRT-PCR analysis	
UN00434-F	5'-CCGCAGCAACAACGTCAAAT-3'
UN00434-R	5'-TACCCAAATTACCCGCACCC-3'
UN04346-F	5'-CACCGAGAACGGAGAAGCAA-3'
UN04346-R	5'-GGGGCTCAGGAGCATAAACA-3'
UN22083-F	5'-AGCGGAGTCCTCAGTCCAAT-3'
UN22083-R	5'-CCTGAACCGTATCCTTCGCA-3'
UN58496-F	5'-GGACGACAAGGCGAGAATGA-3'
UN58496-R	5'-TGTTGCGTCCTACCCATCAC-3'
UN00688-F	5'-GTGTATGGGTCTGGATCGGG-3'
UN00688-R	5'-CAGACCCGGGATAAACCGAA-3'
UN03646-F	5'-AGCTCGAGTCTTGTCTTTCAACT-3'
UN03646-R	5'-AACTCACAGCTTGACAGCGT-3'
UN55363-F	5'-AGGTTCGTATCTGCAGGGTT-3'
UN55363-R	5'-TTGCCGATGTGTCAAAACGC-3'
CmNF-YB8 (UN69086)-F	5'-GGGCAATGGCTACACTTGGT-3'
CmNF-YB8 (UN69086)-R	5'-TCATTCCCTGTGCGTTTGGA-3'
CmSPL3-F	5'-AGCCATGGAGGCCACTTTTT-3'
CmSPL3-R	5'-GTGGACGTCAACAGCGTTTC-3'
CmSPL5-F	5'-GAATACCCGCAACGACAACAG-3'
CmSPL5-R	5'-GTTGCAAGGTGTTGCCAGG-3'
CmSPL9-F	5'-CCACCAACTGGTGACAACCC-3'

CmSPL9-R	5'-GGGTGGTTCAAGTGGCTCT-3'
CmUBI3-F	5'-AGCTGAGCAGACTCCCGATG-3'
CmUBI3-R	5'-AGGCGAATCATCAGTACCAAGT-3'
cmo-miR156-F	5'-TGACAGAAGAGAGTGAGCAC-3'
AtmiR156F	5'-ACACTCCAGCTGGGTGACAGAAGA-3'
Stemloop universal-R	5'-AACTGGTGTCGTGGAG-3'
U6-F	5'-GATAAAATTGGAACGATACAG-3'
U6-R	5'-ATTTGGACCATTTCTCGATTT-3'
miR156 stemloop primer	5'-CTCAACTGGTGTCGTGGAGTCCGGCAATTCAGTTGAGGTGCTCAC-3'
Pri-cmo-MIR156-F	5'-AAAGAGGAAAGCTGCACATTAGG-3'
Pri-cmo-MIR156-R	5'-GCTTCAAGCATATCAAACTATTTGC-3'
For modified 5' RLM-RACE	
CmSPL3-inner-R	5'-GCCGATTATTTATACGTAAAGGACA-3'
CmSPL3-outer-R	5'-AGAAGGTGTGTTTTTATTAGCAATG-3'
CmSPL5-inner-R	5'-AATATTGCATTACATCATTCGACACAAC-3'
CmSPL5-outer-R	5'-ATGCAAACACTTAGAAGTTAACATTCAA-3'
CmSPL9-inner-R	5'-AGAAGAGTCATAACGCCTTCCAC-3'
CmSPL9-outer-R	5'-TGTCGCAACAAAACACACATAAT-3'
For cloning *cmo-MIR156* primary transcript	
Cmo-miR156-GST1-F	5'-ACTCAATAGTGTGTGTTCCAGTGCT-3'
Cmo-miR156-GST2-F	5'-AAAGAGGAAAGCTGCACATTAGG-3'
Cmo-miR156-GST1-R	5'-TCTTCTGGGCTGATGCTTATGTG-3'
Cmo-miR156-GST2-R	5'-CCCTAATGTGCAGCTTTCCTCT-3'
For ChIP-qPCR	
cmo-miR156-P0-F	5'-AAAGAGGAAAGCTGCACATTAGG-3'
cmo-miR156-P0-R	5'-GCTTCAAGCATATCAAACTATTTGC-3'
cmo-miR156-P1-F	5'-CGCTGATTTCATAACCCGCTCT-3'
cmo-miR156-P1-R	5'-TCACGTTACTAGCTTCTTAGATCGT-3'
cmo-miR156-P2-F	5'-GATCTAAGAAGCTAGTAACGTGAATGT-3'
cmo-miR156-P2-R	5'-GGCAACCCATCAATTCAATCAT-3'
cmo-miR156-P3-F	5'-TGATTGAATTGATGGGTTGCC-3'
cmo-miR156-P3-R	5'-AGTCATTATATGTGCAGCCATTCTT-3'
cmo-miR156-P4-F	5'-CACATTTCATGTGCAGCGAAC-3'
cmo-miR156-P4-R	5'-CGACTTCTACGGTTCTACCATGA-3'
cmo-miR156-P5-F	5'-CATGGTAGAACCGTAGAAGTCGATC-3'
cmo-miR156-P5-R	5'-ACCTAACAACCAACCTATTAACGCA-3'
cmo-miR156-P6-F	5'-TAATAGGTTGGTTGTTAGGTCTGAT-3'
cmo-miR156-P6-R	5'-GAAGATTTCAAGTCAAAGTAAGCCT-3'
cmo-miR156-P7-F	5'-CAGGCTTACTTTGACTTGAAATCTT-3'
cmo-miR156-P7-R	5'-TTAGAATACTCACAAGACGCACAAG-3'
AtmiR156a-F1	5'-CGATCAGCGTAATTTTAAGTGAAGC-3'
AtmiR156a-R1	5'-TCGTGTAGTTGAGTCAAAGGACACC-3'
AtmiR156a-F2	5'-ACCCCGATTTTGATGAGCAC-3'
AtmiR156a-R2	5'-AATCATATCCAACCATAAACGGTCT-3'
AtmiR156a-F3	5'-ACCACCACATTGTCTGTCCC-3'
AtmiR156a-R3	5'-GGGTGAAACTTCCGGACTCG-3'
AtmiR156a-F4	5'-ACTCGAGTCCGGAAGTTTCAC-3'
AtmiR156a-R4	5'-GGATGTGAGCAAGATGACATAGA-3'
AtmiR156c-F1	5'-TGCAATGGGACAGATGTCTGAA-3'
AtmiR156c-R1	5'-GGCTTGTCGTTGCCGTTTAT-3'
AtmiR156c-F2	5'-AGCAAACAAACACAAATTAACCGA-3'
AtmiR156c-R2	5'-GCAAAAAGGACAAAGTATCTGGG-3'
AtmiR156c-F3	5'-ACCACCAATTTCACCCAGATAC-3'
AtmiR156c-R3	5'-TCAGCTTATTCTCAGCATTTTGCAC-3'
AtActin2-F	5'-ACCACCAATTTCACCCAGATAC-3'
AtActin2-R	5'-GCTGAGGGATGCAAGGATTGATC-3'
